# Big mountains but small barriers: Population genetic structure of the Chinese wood frog (Rana chensinensis) in the Tsinling and Daba Mountain region of northern China

**DOI:** 10.1186/1471-2156-10-17

**Published:** 2009-04-09

**Authors:** Aibin Zhan, Cheng Li, Jinzhong Fu

**Affiliations:** 1 Department of Integrative Biology, University of Guelph, Guelph, Ontario, N1G 2W1, Canada; 2 Chengdu Institute of Biology, Chinese Academy of Sciences, Chengdu, 410 061, PR China

## Abstract

**Background:**

Amphibians in general are poor dispersers and highly philopatric, and landscape features often have important impacts on their population genetic structure and dispersal patterns. Numerous studies have suggested that genetic differentiation among amphibian populations are particularly pronounced for populations separated by mountain ridges. The Tsinling Mountain range of northern China is a major mountain chain that forms the boundary between the Oriental and Palearctic zoogeographic realms. We studied the population structure of the Chinese wood frog (*Rana chensinensis*) to test whether the Tsinling Mountains and the nearby Daba Mountains impose major barriers to gene flow.

**Results:**

Using 13 polymorphic microsatellite DNA loci, 523 individuals from 12 breeding sites with geographical distances ranging from 2.6 to 422.8 kilometers were examined. Substantial genetic diversity was detected at all sites with an average of 25.5 alleles per locus and an expected heterozygosity ranging from 0.504 to 0.855, and two peripheral populations revealed significantly lower genetic diversity than the central populations. In addition, the genetic differentiation among the central populations was statistically significant, with pairwise *F*_*ST *_values ranging from 0.0175 to 0.1625 with an average of 0.0878. Furthermore, hierarchical AMOVA analysis attributed most genetic variation to the within-population component, and the between-population variation can largely be explained by isolation-by-distance. None of the putative barriers detected from genetic data coincided with the location of the Tsinling Mountains.

**Conclusion:**

The Tsinling and Daba Mountains revealed no significant impact on the population genetic structure of *R. chensinensis*. High population connectivity and extensive juvenile dispersal may account for the significant, but moderate differentiation between populations. Chinese wood frogs are able to use streams as breeding sites at high elevations, which may significantly contribute to the diminishing barrier effect of mountain ridges. Additionally, a significant decrease in genetic diversity in the peripheral populations supports Mayr's central-peripheral population hypothesis.

## Background

Amphibians in general are poor dispersers and highly philopatric with strict habitat specificity and physiological requirements [1,2]. Pond breeding amphibians are particularly so because they need specific, sometimes distinct, habitats for breeding and larval development [3,4]. Consequently, significant population genetic structure is expected, especially over moderately large geographic distances or when landscapes are fragmented [4]; this expectation has been supported by numerous phylogeographic and population genetic studies. For example, most phylogeographic analyses based on mitochondrial DNA found significant differentiation among different amphibian populations [2]. Substantial population genetic structure in amphibians has also been revealed using nuclear DNA markers. For example, using microsatellite DNA data, Rowe et al. (2000) found an average *F*_*ST *_value of 0.224 in a toad species (*Bufo calamita*) among populations separated by less than 16 kilometers [5].

Genetic differentiation among amphibian populations appears to be relatively high for populations separated by mountain ridges [6-11]. Using microsatellite DNA loci, Funk et al.'s (2005) study of the Columbian spotted frog (*Rana luteiventris*) highlighted the impact of mountain ridges on amphibian population structure [10]. They found significantly reduced gene flow between populations situated on either side of mountain ridges and between low- and high-elevation populations, despite close geographic proximity. They also found reduced genetic diversity within high-elevation populations. Variation in selection regimes and physiological limits of amphibian species were suggested to have contributed to the observed differentiation [10]. Evidence for such impact also comes from biogeographic studies showing that the distributions of many amphibians are bounded by mountain ridges [12].

The Tsinling Mountain range in northern China is one of the few major mountains on Earth that extend from east to west. With the combined altitudinal and latitudinal differences, a great reduction in gene flow across the range is expected. Its peak, Mt. Taibai, reaches 3767 meters above sea level (a.s.l.) and most parts of the ridge are higher than 1800 meters a.s.l. The range divides two major water drainages in East Asia, the Yellow River system to the north and the Yangtze River system to the south. The fauna at the northern and southern sides of the Tsinling Mountains is so different that the Tsinling is recognized as the boundary between the Oriental and Palearctic zoogeographic realms [13]. Approximately 50 kilometers to the south, the Daba Mountains (including the Micang Mountains) extend from east to west in parallel with the Tsinling Mountains. The Hanzhong Plain lies between the two mountain ranges. To a lesser degree, the Daba Mountains also form a significant geographical divider of the local fauna [14].

**Figure 1:**
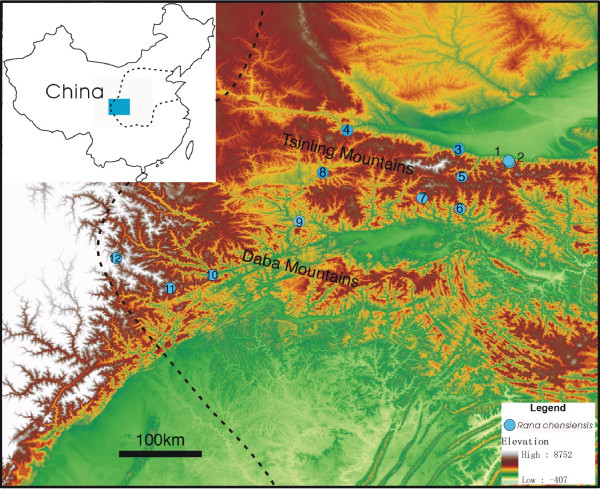
**Sampling sites of the Chinese wood frog (*Rana chensinensis*)**. Sampling site names are listed in Table 1. Sites 1–5 are located at the northern side of the Tsinling; sites 6–9 are located at the southern side of the Tsingling, which is also the northern side of the Daba; sites 10–12 are located at the southern side of the Daba. Sites 11 and 12 are located at the periphery of the species' distribution. Insert: outline of China; the blue block indicates the relative location of the studying area and the dashed line indicates the species' distribution.

The Chinese wood frog (*Rana chensinensis*) is a common species in central and northern China with a wide distribution (latitude: N31° to N42°, elevation: 300 to 3100 meters a.s.l.; Figure 1) [15]. These frogs primarily breed in ponds, particularly at low elevations, but are capable of using slow moving water bodies of mountain streams as breeding sites [16,17]. The breeding season lasts 3 – 4 weeks in late March to early April, and their tadpoles reach metamorphosis within two months [16,17]. Several major mountains such as the Tsinling Mountains and the Daba Mountains are located within its distribution range, which makes *R. chensinensis *an excellent model to study the impact of major landscape features, particularly mountains, on amphibian population genetic structure.

Using 13 polymorphic microsatellite DNA markers, we examined the population genetic structure of the Chinese wood frog from 12 breeding sites in the Tsinling-Daba Mountain region over a large geographical distance. Our objective was to test the hypothesis that mountain ridges provide significant barriers to gene flow in amphibian species [10]. We predicted that populations of the Chinese wood frog would have significant levels of genetic differentiation, and form three distinct groups, one at the north of the Tsinling Mountains, one between the Tsinling and Daba Mountain chains, and one at the south of the Daba Mountains. Populations from the same side of the mountain ridges should have similar genetic composition, while populations from different sides should have large amounts of genetic differentiation.

## Results

### Variation within sites

A total of 523 individuals from 12 breeding sites were examined (Figure 1, Table 1). Genetic diversity indices for each site are presented in Additional file 1. All 13 microsatellite DNA loci were polymorphic across all 12 sites. The number of alleles per locus ranged from 14 (RCMS011 and RCMS026) to 47 (RCMS028) among all sites. For all loci across all sites, a total of 332 different alleles were detected, with an average of 25.5 alleles per locus. The allelic richness of each site ranged from 4.1 to 12.2 and the average expected heterozygosity ranged from 0.504 to 0.855. While most loci conformed to HWE, 39 cases out of a total 156 cases of tests showed significant deviations after sequential Bonferroni corrections. However, no locus or site had a particularly high number of sites/loci that deviated from HWE (see Additional file 1). Most of the deviated cases showed a significant heterozygote deficiency (see Additional file 1), suggesting the possible presence of a null allele(s).

**Table 1:** Collection information for the Chinese wood frog (*Rana chensinensis*)

Site number	Sample locality	Elevation (a.s.l.)	Coordinates	*N*
1	Shangyinjiapo, Laoyu Subco, Hu Co, Shaanxi Province	1600 m	N33.98795° E108.50748°	37
2	Xiayinjiapo, Laoyu Subco, Hu Co, Shaanxi Province	688 m	N33.96634° E108.51907°	26
3	Tangyu, Meixian Co, Shaanxi Province	560 m	N34.18070° E107.82583°	50
4	Shigu, Baoji City, Shaanxi Province	775 m	N34.29952° E107.14037°	50
5	Banfangzi, Zhouzhi Co, Shaanxi Province	1149 m	N33.80635° E107.99083°	35
6	Xichahe, Foping Co, Shaanxi Province	1100 m	N33.45655° E107.96894°	48
7	Huayang, Yang Co, Shaanxi Province	1100 m	N33.57588° E107.55029°	45
8	Fengxian, Fengxian Co, Shaanxi Province	954 m	N33.90088° E106.53301°	50
9	Lueyang, Lueyang Co, Shaanxi Province	816 m	N33.26540° E106.24962°	50
10	Bikou, Wen Co, Gansu Province	687 m	N32.72133° E105.22530°	32
11	Tangjiahe, Qingchuan Co, Sichuan Province	1442 m	N32.57795° E104.75372°	50
12	Wanglang, Pingwu Co, Sichuan Province	2480 m	N32.90927° E104.15594°	50

*N *= sample size

Two sites (11 and 12), which are located at the periphery of the species' distribution (Figure 1), had substantially lower estimates of genetic diversity than the central populations (1–10). For site 11, both the estimated average allelic richness and the expected level of heterozygosity were significantly lower than for any of the ten central sites with only one exception (Mann-Whitney U test with sequential Bonferroni correction; Table 2). Though not as strong, similar patterns were seen for site 12, which had statistically significantly lower estimates of average allelic richness and heterozygosity than half of the ten central sites (Mann-Whitney U test with sequential Bonferroni correction; Table 2).

**Table 2:** *P*-values for the exact test of difference in allelic richness (below diagonal) and expected heterozygosity (above diagonal) using a non-parametric test (Mann-Whitney U test).

Site	1	2	3	4	5	6	7	8	9	10	11	12
1	-----	0.891	0.312	0.059	0.082	0.239	0.098	0.392	0.892	0.404	**< 0.001**	0.002
2	0.537	-----	0.198	0.183	0.301	0.398	0.113	0.491	0.899	0.309	**< 0.001**	0.014
3	0.388	0.361	-----	0.381	0.372	0.100	0.059	0.902	0.409	0.118	**< 0.001**	**< 0.001**
4	0.281	0.211	0.400	-----	0.194	0.201	0.190	0.592	0.113	0.102	**< 0.001**	**< 0.001**
5	0.452	0.092	0.069	0.007	-----	0.623	0.389	0.223	0.223	0.903	**< 0.001**	0.287
6	0.923	0.981	0.291	0.063	0.059	-----	0.409	0.121	0.620	0.861	**< 0.001**	**< 0.001**
7	0.472	0.194	0.031	0.015	0.401	0.081	-----	0.059	0.099	0.303	0.030	0.503
8	0.194	0.052	0.701	0.798	0.104	0.102	**0.001**	-----	0.291	0.101	**< 0.001**	**< 0.001**
9	0.402	0.312	0.780	0.099	0.084	0.210	0.100	0.423	-----	0.444	**< 0.001**	0.009
10	0.092	0.119	0.009	0.009	0.901	0.083	0.582	**< 0.001**	0.079	-----	**< 0.001**	0.305
11	**< 0.001**	**< 0.001**	**< 0.001**	**< 0.001**	**< 0.001**	**< 0.001**	**< 0.001**	**< 0.001**	**< 0.001**	**< 0.001**	-----	**< 0.001**
12	**< 0.001**	0.014	**< 0.001**	**< 0.001**	0.099	0.003	0.401	**< 0.001**	**< 0.001**	0.032	0.104	-----

Bold numbers indicate statistical significance after sequential Bonferroni corrections.

### Differentiation among sites

Pairwise estimates of *F*_*ST *_varied from 0.0175 to 0.3130, and all were statistically significant, indicating differentiation between population pairs (see Additional file 2). The pairwise *F*_*ST *_values among the ten central sites varied from 0.0175 to 0.1625 with an average of 0.0878, indicating low to moderate differentiation among these sites. The pairwise *F*_*ST *_between the peripheral sites (11 and 12) and the central sites were generally higher, ranging from 0.1206 to 0.3130 (see Additional file 2).

The estimated migration rates between sites are listed in Additional file 2. The number of migrants per generation ranged from 0.11 to 3.24, and several site pairs separated by mountains and large geographical distances had migration rates close to or higher than 1.00. The highest value was observed between sites 1 and 2, which was not surprising because the two sites are only 2.6 kilometers apart. The migration rates between sites sampled from different sides of the mountains were not substantially lower than others (see Additional file 2).

**Figure 2:**
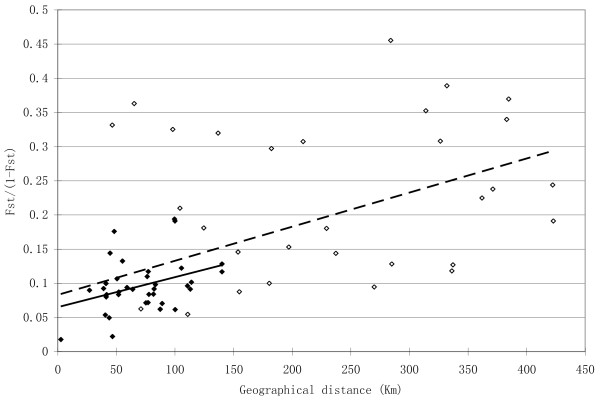
**Correlation between geographical distance in kilometers (x-axis) and genetic distance given as *F*_*ST*_/(1-*F*_*ST*_) values (y-axis)**. The empty diamonds represent the site-pairs among all sampling sites and the solid diamonds represent the site-pairs among the nine sites immediately north or south of the Tsinling range. The dashed line represents the linear regression using data derived from all sites and the solid line represents the linear regression using data derived from the nine Tsinling sites (sites 1 – 9).

A linear regression of *F*_*ST*_/(1 - *F*_*ST*_) values against geographical distance (in kilometers) is presented in Figure 2. When data from all twelve sites were included, a weak but significant positive correlation between these two variables was observed (*P*_*Mantel *_< 0.001, *R*^2 ^= 0.3116). The two peripheral populations (11 and 12) had reduced genetic diversity and thus high pairwise *F*_*ST *_values with the central populations, and they also had large geographical distances from the central populations. Therefore, including them in the analysis might have artificially enhanced the correlation. To further test the impact of the Tsinling Mountains and eliminate the potential impact of the peripheral populations, a second IBD analysis that included only the nine Tsinling sites was conducted. It also showed a weak but significant positive correlation (*P*_*Mantel *_< 0.001, *R*^2 ^= 0.2279, Figure 2).

### AMOVA and putative genetic barrier prediction

The twelve sites were divided into three groups for AMOVA under the hypothesis that the Tsinling and Daba Mountains are two major genetic barriers. Sites from the same side of a mountain chain were grouped together, i.e. Group I = sites 1–5, Group II = sites 6–9, and Group III = sites 10–12 (Figure 1). The hierarchical multi-locus AMOVA including all sites indicated that the largest percentage of variation (85.63%) was attributed to the among-individuals-within-sites component and a small portion of the genetic variance (10.12%) was attributed to the among-sites-within-groups component. Only 4.26% of the variance was attributed to the among-groups component (Table 3). To minimize the impact of the reduced genetic diversity of the two peripheral sites, a second AMOVA test that included only the ten central sites with the same grouping was conducted and resulted in a similar distribution of variation (Table 3).

**Table 3:** Results of the analysis of molecular variance (AMOVA).

A	Source of variation	Sum of square	Variance components	Percentage variation	*P*-value
	
	Among groups	272.039	0.24305	4.25577	< 0.00001
	Among sites within groups	478.176	0.57788	10.11878	< 0.00001
	Within populations	4899.441	4.89003	85.62546	< 0.00001
	Total	5649.656	5.71096		
B	Source of variation	Sum of square	Variance components	Percentage variation	*P*-value
	
	Among groups	147.848	0.16706	2.91249	< 0.00001
	Among sites within groups	266.329	0.39323	6.85549	< 0.00001
	Among individuals within sites	4202.064	5.17572	90.23202	< 0.00001
	Total	4616.242	5.73601		

Sites are separated into three groups under the hypothesis that the Tsinling and Daba Mountains present significant barriers to gene flow. A: analysis includes all 12 sites; group 1 = sites for north of the Tsinling Mountains (1, 2, 3, 4 and 5), and group 2 = sites from south of the Tsinling Mountains (6, 7, 8 and 9), and group 3 = sites from south of the Daba Mountains (10, 11 and 12). B: analysis includes only the 10 central sites; group 1 = sites 1, 2, 3, 4 and 5, group 2 = sites 6, 7, 8 and 9, and group 3 = site 10.

**Figure 3:**
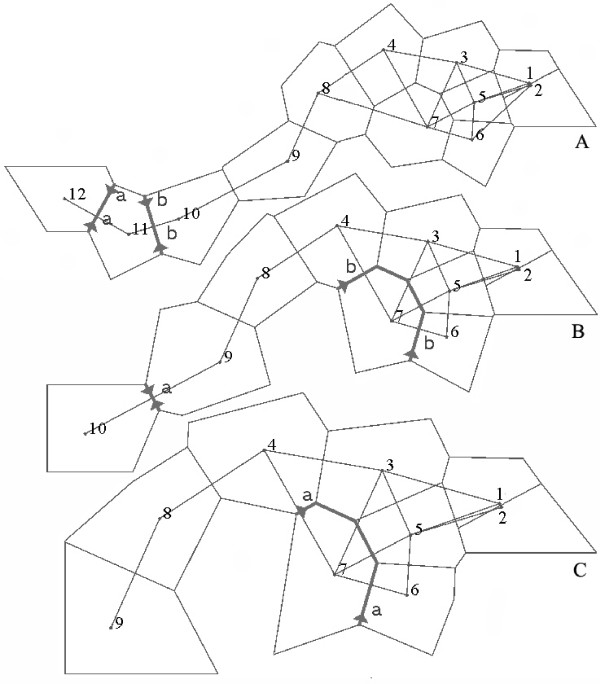
**Genetic barriers predicted by BARRIER (version 2.2)**. The genetic barriers are shown in bold lines with arrows. A: genetic barrier predication using the data from all sites (1 – 12); B: genetic barrier predication using the data from the ten central populations (1 – 10); C: genetic barrier prediction using the data from the nine Tsinling populations (1 – 9).

The barrier prediction analysis using Monmonier's maximum difference algorithm identified two putative barriers when all sites were included (Figure 3A). The first barrier separated peripheral site 12 from all other sites. The second predicted barrier separated the second peripheral site, 11, from the central sites. When only the ten central sites were included, again two barriers were detected. The first separated site 10 from the other nine sites, and may reflect a weak barrier effect imposed by the Daba range, while the second separated site 7 from all the rest (Figure 3B). When only the nine Tsinling sites were included in the analysis, one barrier was detected, which separated site 7 from the other sites (Figure 3C). None of the putative barriers detected from genetic data coincided with the location of the Tsinling Mountains (Figure 3).

## Discussion

### The Tsinling and Daba Mountains do not present significant genetic barriers to the Chinese wood frog

A high level of genetic diversity was detected at microsatellite DNA loci in Chinese wood frogs. Two commonly used indices of genetic diversity, allelic diversity and heterozygosity, are comparable to or higher than those found in other similar studies of anurans. For example, Funk et al. (2005) found 5 – 16 alleles per locus and expected heterozygosity of 0.23 – 0.66 in various populations of the Columbian spotted frog (*Rana luteiventris*) [10], while Johansson et al. (2006) found 10 – 34 alleles per locus (mean = 19) in *Rana temporaria *across Sweden [18]. We found 14 – 47 alleles per locus (mean = 25.5) and *H*_*E *_of 0.504 to 0.855 in different populations.

Low to moderate genetic differentiation between the ten central sites was observed, with pairwise *F*_ST _ranging from 0.0175 to 0.1625, and with an average of 0.0878 (see Additional file 2). Considering the large geographical distance (up to 337.1 km) and two large mountain chains within the region, it is somewhat surprising that greater population subdivision was not found, as these values are much lower than other studies at similar or even smaller geographical scales. Using microsatellite DNA markers, Funk et al. (2005) reported *F*_*ST *_values of 0.153–0.242 between Columbian spotted frog populations (*Rana luteiventris*) from different sides of mountain ridges within distance of only 10 kilometers [10]. Furthermore, Spears et al. (2005) reported pairwise *F*_ST _values up to 0.453 between populations within a major mountain valley and a distance of 60 kilometers in tiger salamanders (*Ambystoma tigrinum*) [11]. While higher *F*_*ST *_values were observed when the two peripheral sites were also included (up to 0.3130), these values may have been inflated by the low genetic diversity of the peripheral sites.

A moderate to high level of migration is the most likely explanation for the low to moderate differentiation between sites. Despite geographical distances of 50 – 100 kilometers and a major mountain range between them, the migration rates between site-pairs from different sides of the Tsinling Mountains ranged from 0.17 to 1.26 individuals per generation with an average of 0.79 (immigration rate; Additional file 2). Studies of migration rate in other anurans have usually found much lower values. For example, Stevens et al. (2006) reported *N*_*m *_of 0.03 to 0.34 between sites within distance of 15 kilometers for the natterjack toad (*Bufo calamita*) [19]. Additionally, the large allele numbers of these microsatellite DNA loci may partially contribute to the relatively low *F*_*ST *_values. Hedrick (1999) analytically demonstrated that, when the amount of within-population variation becomes high, the *F*_*ST *_values necessarily become low [20]. Other evolutionary processes, such as recent population colonization, may also contribute to the low to moderate differentiation between sites. However, the Chinese wood frog populations of the Tsinling and Daba region are unlikely to have been recently established, as recently established populations typically have low levels of genetic diversity [21] whereas these wood frog populations have very high genetic diversity (see Additional file 1). Furthermore, the Tsinling region was not covered by ice during Pleistocene glaciations [22], and therefore, there was no post-glaciation colonization.

The Tsinling and Daba Mountain ranges do not present significant barriers to gene flow in Chinese wood frogs. Both AMOVA and Monmonier's maximum difference algorithm have proven to be powerful tools for detecting the impact of landscape features on population genetic structure [3,23]. Nevertheless, our AMOVA attributed most variation to the within-site component (Table 3). Furthermore, much of the variation among sites can be explained by isolation-by-distance, as the correlation between geographical and genetic distance was very strong (Figure 2). None of the predicted genetic barriers concurred with the Tsinling Mountains (Figure 3). In only one case did a predicted genetic barrier correspond to the location of the Daba Mountains, when only the ten central populations were included (Figure 3B). When all populations were included, however, the association disappeared (Figure 3A). The Daba Mountains may impose a weak genetic barrier to the populations of the Chinese wood frog. Whether the Tsinling and Daba Mountains present significant barriers (or not) to other species is an interesting topic for future study.

These results prompt the question: Given that mountains have repeatedly been shown to have strong effects on gene flow in amphibians, how did a major geologic divider such as the Tsinling Mountains not impose any significant genetic barrier to the Chinese wood frog? Large gene flow among neighboring sites (high connectivity) is probably the primary cause of the diminished mountain effect, and extensive juvenile dispersal may be responsible for such gene flow. Most mature female Chinese wood frogs produce 400 to 900 eggs annually, with some producing up to 2000 eggs, which is higher than other closely related wood frog species [16,24]. Thus, Chinese wood frogs have the capacity to produce a very large number of juveniles. Although a high ratio of mortality was observed at larval or juvenile stages, the number of emerging juveniles is still extremely large (Fu, personal observation). With a large number of juveniles, even a small percentage of successful migrants will create sufficient gene flow to counter population differentiation. Furthermore, there are many available breeding sites between our sampling sites, which may facilitate the dispersal. The relatively high *N*_*m *_values in this study support this explanation (see Additional file 2). In a closely related and biologically similar species, Brede & Beebee (2004) also found high connectivity among subpopulations of *R. temporaria *[25], although other studies revealed substantial population structure [26]. Funk et al(2005) also found a large number of juvenile Columbia spotted frogs dispersing a large distance (> 5 km) [27].

Chinese wood frogs are capable of using slow moving mountain streams as breeding sites at high elevations and are not restricted to breeding in ponds. For example, at one of our collecting sites, site 12, the frogs bred in a stream at an elevation of 2480 meters a.s.l. This life history trait is perhaps crucial for the Chinese wood frog to maintain a low to moderate population differentiation with the presence of major mountain ridges. Mountain ridges have significant impact on pond breeder population structure, which is well established by numerous studies [6-11]. Nevertheless, whether the same impact occurs on stream breeders is still an open question. Additionally, the Chinese wood frog may use mountain passes and valleys as corridors to cross the seemingly impossible high mountain ridges. Lu et al. (2006) reported that the Chinese wood frog hibernates at an elevation of 2000 meters a.s.l. [17]. There are many mountain passes lower than 2000 meters a.s.l. along the chain of Tsinling Mountains.

### Low genetic diversity of the peripheral populations

Populations at the periphery of a species' distribution tend to have lower genetic diversity than the central populations [28], and Lesica & Allendorf (1995) attributed suboptimal habitat, greater isolation, founder effects and/or genetic bottleneck as the factors that would decrease the diversity at the periphery [29]. Results from empirical studies are mixed [30,31]. The two peripheral populations (11 and 12) examined in this study clearly demonstrated a significant decrease in both allele richness and expected heterozygosity (Table 2 & Additional file 1). No catastrophic events (such as a forest fire) have been reported in the past 50 years in these two areas, which would likely lead to a bottleneck and/or a founder effect. We are not aware of any other obvious reasons that these populations should have reduced genetic diversity. Rather, suboptimal environments in peripheral regions may best explain the observed reduction. The two populations are located at high elevations where development rates are retarded and sexual maturity is delayed compared to populations at lower elevations [17]. Additionally, peripheral populations tend to have smaller population sizes than central populations [31,32], which can also lead to a reduction of genetic variation. Funk et al. (2005) also found low diversity for high elevation populations, which they attributed to small population sizes [10].

## Conclusion

Our study presents an exception to the currently accepted hypothesis that mountain ridges impose major barriers to amphibian movement. The Tsinling Mountains surprisingly demonstrated no statistically detectable barrier to the gene flow among populations of the Chinese wood frog (*Rana chensiensis*). Some amphibian species, particularly those with large numbers of juveniles, may have high population connectivity and hence be able to maintain relatively large gene flow across natural or man-made geographical barriers. Mountain ridges likely only impose a weak barrier effect to stream breeders. Life history traits and properties related to population genetic structure should be carefully evaluated before using amphibian species to assess environment quality, such as habitat fragmentation [25].

## Methods

### Sampling

Samples from twelve breeding sites were collected from the Tsinling and Daba Mountain region. Eight (1, 2, 5, 6, 7, 10, 11, and 12) were collected between the 1^st ^and 13^th ^of April, 2007 and the other four (3, 4, 8 and 9) were collected between the1^st ^and 10^th ^of April, 2008. The Chinese wood frog typically takes two (male) to three (female) years to reach sexual maturity [17] and the observed census population size in this region is very large. Therefore, we assumed that population genetic variation within one year is minimal and such variation was ignored in our analysis. Sampling sites were selected from both sides of the Tsinling Mountains and the Daba Mountains (Figure 1). Five sites (1–5) are located at the northern side of the Tsinling; four sites (6–9) are located at the southern side of the Tsingling, which is also the northern side of the Daba; three sites (10–12) are located at the southern side of the Daba. Of the 12 sites, 11 and 12 are located at the periphery of the species' distribution, while the others are relatively central. The linear geographical distance among the sites ranges from 2.6 to 422.8 kilometers with an average of 147.1 kilometers.

Adult frogs were collected from a single pond at each site during their breeding season. Two toes from two limbs were clipped from each individual and the toe samples were preserved in 95% ethanol. Ten individuals from each site were euthanized and preserved as reference voucher specimens. All other frogs were immediately released at the point of capture after toe clipping. The toes from a total of 523 individuals were collected for this study and the detailed sample site and sample size information is provided in Table 1. All collecting procedures and permits followed the approved guidelines by the Chengdu Institute of Biology.

### Microsatellite DNA analysis

Total genomic DNA was isolated from the toe samples using the standard phenol/chloroform method. Polymerase chain reaction (PCR) amplifications were performed using thirteen (RCMS007, RCMS009, RCMS010, RCMS011, RCMS026, RCMS028, RCMS029, RCMS030, RCMS035, RCMS042, RCMS092 and RCMS107) polymorphic microsatellite DNA markers developed by Zhan & Fu (2008) [33]. One primer of each primer pair was labeled with tetrachloro-6-carboxy-fluorescine (TET). A total of 10 μL PCR mix contained 20 – 40 ng of genomic DNA, 1 × ThermoPol™ Buffer (2 mM Mg^2+^, New England Biolabs), 200 μM of each dNTP, 0.25 μM of each primer and 0.5 U of *Taq *DNA Polymerase™ (New England Biolabs). The PCR cycling parameters were: 95°C for 5 minutes, then repeated 30 times at 95°C for 30 seconds, 52°C for 30 seconds and 72°C for 30 seconds, and a final extension at 72°C for 5 minutes. The PCR products were denatured and electrophoresed on 6% denaturing polyacrylamide gels and visualized on a FMBIO laser scanner (Hitachi). The alleles were scored relative to a TAMRA™ size standard marker (Genescan™ 350, Applied Biosystems) and against the frog sample that was used to design the primers [33].

### Statistical analysis

Genetic diversity within sites was measured with five indices. The number of alleles (*A*) and their frequency (*F*), the observed heterozygosity (*H*_*O*_) and the expected heterozygosity (*H*_*E*_) were calculated using the computer program POPGENE 32 [34] or ARLEQUIN (version 3.1) [35]. The allelic richness (*A*_*r*_) [36], which is the mean number of alleles corrected for population sample size, was calculated using the program FSTAT [37]. In addition, a Markov chain method [38] was employed to estimate the probability of significant deviation from Hardy-Weinberg equilibrium (HWE) using the online version of the program GENEPOP () [39]. Significance criteria were adjusted for the number of simultaneous tests using sequential Bonferroni corrections [40].

The degree of population subdivision was determined from mutilocus estimates of *F*_*ST *_(= *θ*) [41] for all population pairs. *F*_*ST *_values were calculated using ARLEQUIN. Pairwise significance tests for *F*_*ST *_were performed by permutation and resampling of multilocus genotypes among pairs of samples. To ensure small standard deviations, 10000 permutations were performed to allow for the significance at the 1% nominal level after sequential Bonferroni corrections. Additionally, difference in allelic richness (*A*_*r*_) and expected heterozygosity (*H*_*E*_) between sites was also tested using a non-parametric test (Mann-Whitney U test) in SPSS version 12 for Windows. The statistical significance for population pairs was evaluated at the level of 0.05 after sequential Bonferroni correction.

To illustrate the dispersal patterns between breeding sites, the migration rates (*N*_*m*_) between sites were calculated based on multilocus genotypes with Bayesian inference using the program MIGRATE (version 2.4.1) [42]. The number of migrants per generation was calculated as *θ*_*i*_*M*_*i*_, where *θ*_*i *_equals *x**μ *and *M*_*i *_equals *m*_*i*_/*μ*. Among the parameters, *x *is the inheritance parameter;  is the effective population size; *μ *is the mutation rate per locus per generation; and *m*_*i *_is the immigration rate. For our analysis, *x *was set as 4, the value commonly used for nuclear gene data, and other parameters were estimated from the data by the program. A Brownian motion mutation model was used. We used 10 short chains (10,000 iterations) and 3 long chains (1,000,000 iterations) with 50,000 iterations discarded as an initial 'burn-in' for the Bayesian search strategy. The replicates index was set as '= YES: 5' and the randomtree index was set as '= YES'. A static heating scheme was employed with 'heating = ADAPTIVE: 1{1.0 1.5 3.0 6.0}'. The simulation was repeated 5 times to ensure consistency. For an initial run, we used *θ *(*F*_*ST*_) to find the starting parameters. In the subsequent runs, we changed the random number seed and the Bayesian estimates of *θ *and *M *from the previous run as the new starting values. The results from the separated runs were nearly identical and the estimates from the last run were used.

Isolation by distance (IBD) was examined by testing the correlation between Rousset's (1997) *F*_*ST*_/(1-* F_ST_*) [43] and geographical distance using the Mantel test. The linear distances between sampling sites were estimated using GoogleEarth with their coordinates. For testing of statistical significance, 10000 permutations in Mantel tests were used to test the null hypothesis that genetic distance and geographical distance are independent. The Mantel tests were carried out using GENEPOP.

The impact of mountains on population structure was tested with a locus by locus analysis of molecular variance (AMOVA) [44] using ARLEQUIN. The twelve sites were divided into three groups under the hypothesis that the Tsinling and Daba Mountains are two major genetic barriers: Group I included five sites, 1–5, which are located at the northern side of the Tsinling Mountains; Group II included four sites, 6–9, sampled from the southern side of the Tsinling Mountains and the northern side of the Daba Mountains; and Group III included three sites, 10–12, from the southern side of the Daba Mountains. Permutation tests were performed at three hierarchical levels: among groups, among sites within groups and among individuals within sites.

We also employed Monmonier's maximum difference algorithm [45] to highlight geographical features that are corresponding to pronounced genetic discontinuity using the program BARRIER (version 2.2) [45]. Geographical coordinates were used for each sample and connected by Delauney triangulation using a pairwise *F*_*ST *_genetic matrix. Putative genetic boundaries were identified across the geographical landscapes [45]. The data derived from all sites, ten central sites, and the nine Tsinling sites were analyzed separately to detect if the two major mountain chains correspond to putative barriers of gene flow among the sites.

## Authors' contributions

AZ performed most of the lab work, data analyses and manuscript preparation. CL collected most of the specimens. JF conceived the project. All authors contributed equally to this work in discussing research strategy and development. All authors read and approved the final manuscript.

## Supplementary Material

Additional File 1 **Genetic diversity at 13 microsatellite loci for 12 sites of the Chinese wood frog (*Rana chensinensis*)**. The file provided the original data for sample sizes, number of alleles, allele richness, observed heterozygosity, expected heterozygosity, exact *P*-values for Hardy-Weinberg equilibrium tests. Click here for file

Additional File 2 **Estimates of population genetic differentiation (pairwise *F*_*ST*_) and migration rate between sites**. The file provided the original data of the pairwise *F*_*ST *_and estimates of numbers of immigrants and numbers of emigrants between sites. Click here for file
