# Supplementary material for: Big mountains but small barriers: Population genetic structure of the Chinese wood frog (Rana chensinensis) in the Tsinling and Daba Mountain region of northern China
Source: BMC Genet. 2009 Apr 9;10:17. doi: 10.1186/1471-2156-10-17 (PMC2679764; doi:10.1186/1471-2156-10-17)
Supplement: Additional File 1 — Genetic diversity at 13 microsatellite loci for 12 sites of the Chinese wood frog (Rana chensinensis). The file provided the original data for sample sizes, number of alleles, allele richness, observed heterozygosity, expected heterozygosity, exact P-values for Hardy-Weinberg equilibrium tests. [file 1471-2156-10-17-S1.doc]

**Additional file 1**

Genetic diversity at 13 microsatellite loci for 12 sites of the Chinese wood frog (*Rana chensinensis*). *N*, sample size; *A*, number of alleles; *A_r_,* allele richness; *H_O_*, observed heterozygosity; *H_E_*, expected heterozygosity; *F_IS_* value; *P_HW_*, exact *P*-value for Hardy-Weinberg equilibrium test.

| Sites |  | RCM  S007 | RCM  S009 | RCM  S010 | RCM  S011 | RCM  S026 | RCM  S028 | RCM  S029 | RCM  S030 | RCM  S035 | RCM  S042 | RCM  S092 | RCM  S098 | RCM  S107 | Average |
| --- | --- | --- | --- | --- | --- | --- | --- | --- | --- | --- | --- | --- | --- | --- | --- |
| 1 | *A/A_r_* | 16/14.3 | 10/8.6 | 9/8.5 | 10/9.3 | 10/9.5 | 9/8.3 | 8/7.3 | 9/8.2 | 13/11.9 | 12/11.1 | 9/8.4 | 9/7.9 | 15/12.8 | 10.7/9.7 |
| *N* = 37 | *H_O_* | 0.833 | 0.730 | 0.706 | 0.583 | 0.946 | 0.865 | 0.676 | 0.784 | 0.838 | 0.919 | 0.838 | 0.667 | 0.784 | 0.782 |
|  | *H_E_* | 0.891 | 0.773 | 0.837 | 0.804 | 0.853 | 0.836 | 0.723 | 0.734 | 0.895 | 0.864 | 0.840 | 0.668 | 0.837 | 0.812 |
|  | *F_IS_* | 0.065 | 0.056 | 0.158 | 0.277 | -0.111 | -0.036 | 0.066 | -0.069 | 0.063 | -0.064 | 0.003 | 0.002 | 0.065 | 0.036 |
|  | *P_HW_* | 0.035 | 0.924 | 0.159 | 0.020 | 0.000 | 0.132 | 0.174 | 0.659 | 0.091 | 0.448 | 0.586 | 0.082 | 0.276 | — |
| 2 | *A/A_r_* | 17/17.0 | 6/6.0 | 11/10.9 | 10/10.0 | 10/10.0 | 9/8.9 | 7/7.0 | 10/9.9 | 11/10.8 | 12/11.8 | 6/6.0 | 4/4.0 | 9/9.0 | 9.4/9.3 |
| *N* = 26 | *H_O_* | 0.840 | 0.731 | 0.692 | 0.560 | 0.962 | 0.769 | 0.769 | 0.846 | 0.808 | 0.808 | 0.769 | 0.577 | 0.923 | 0.773 |
|  | *H_E_* | 0.936 | 0.775 | 0.783 | 0.842 | 0.873 | 0.817 | 0.752 | 0.864 | 0.861 | 0.857 | 0.693 | 0.581 | 0.837 | 0.805 |
|  | *F_IS_* | 0.104 | 0.058 | 0.118 | 0.34 | -0.104 | 0.059 | -0.025 | 0.02 | 0.063 | 0.058 | -0.112 | 0.008 | -0.105 | 0.037 |
|  | *P_HW_* | 0.139 | 0.720 | 0.036 | 0.001 | 0.111 | 0.208 | 0.554 | 0.360 | 0.213 | 0.863 | 0.105 | 0.785 | 0.516 | — |
| 3 | *A/A_r_* | 16/13.2 | 8/6.6 | 8/6.5 | 8/7.7 | 8/6.7 | 20/16.2 | 14/11.3 | 13/10.8 | 20/15.8 | 21/17.3 | 12/10.0 | 12/10.3 | 14/11.7 | 13.4/11.1 |
| *N* = 50 | *H_O_* | 0.420 | 0.580 | 0.240 | 0.620 | 0.780 | 0.740 | 0.600 | 0.840 | 0.900 | 0.800 | 0.700 | 0.800 | 0.620 | 0.665 |
|  | *H_E_* | 0.912 | 0.725 | 0.822 | 0.815 | 0.806 | 0.887 | 0.820 | 0.864 | 0.908 | 0.916 | 0.857 | 0.828 | 0.806 | 0.844 |
|  | *F_IS_* | 0.486 | 0.202 | 0.693 | 0.241 | 0.032 | 0.167 | 0.270 | 0.028 | 0.009 | 0.128 | 0.163 | 0.034 | 0.233 | 0.207 |
|  | *P_HW_* | 0.000 | 0.088 | 0.000 | 0.012 | 0.452 | 0.023 | 0.002 | 0.531 | 0.094 | 0.006 | 0.301 | 0.159 | 0.000 | — |
| 4 | *A/A_r_* | 20/14.9 | 12/11.1 | 11/9.6 | 12/9.9 | 9/8.0 | 20/15.3 | 21/17.0 | 11/10.2 | 15/12.3 | 20/15.7 | 12/10.7 | 16/12.2 | 14/12.0 | 14.8/12.2 |
| *N* = 50 | *H_O_* | 0.620 | 0.780 | 0.240 | 0.700 | 0.680 | 0.800 | 0.340 | 0.680 | 0.660 | 0.860 | 0.620 | 0.580 | 0.740 | 0.638 |
|  | *H_E_* | 0.868 | 0.871 | 0.861 | 0.890 | 0.834 | 0.872 | 0.882 | 0.829 | 0.877 | 0.915 | 0.862 | 0.780 | 0.777 | 0.855 |
|  | *F_IS_* | 0.269 | 0.106 | 0.669 | 0.195 | 0.186 | 0.083 | 0.527 | 0.181 | 0.209 | 0.038 | 0.283 | 0.259 | 0.048 | 0.235 |
|  | *P_HW_* | 0.000 | 0.256 | 0.000 | 0.060 | 0.135 | 0.043 | 0.000 | 0.002 | 0.004 | 0.266 | 0.000 | 0.002 | 0.226 | — |

| Sites |  | RCM  S007 | RCM  S009 | RCM  S010 | RCM  S011 | RCM  S026 | RCM  S028 | RCM  S029 | RCM  S030 | RCM  S035 | RCM  S042 | RCM  S092 | RCM  S098 | RCM  S107 | Average |
| --- | --- | --- | --- | --- | --- | --- | --- | --- | --- | --- | --- | --- | --- | --- | --- |
| 5 | *A/A_r_* | 8/8.0 | 7/6.7 | 10/9.6 | 8/7.8 | 9/8.4 | 7/6.7 | 9/8.5 | 8/7.6 | 12/10.8 | 8/8.0 | 5/4.7 | 11/10.6 | 9/8.5 | 8.5/8.2 |
| *N* = 35 | *H_O_* | 0.143 | 0.706 | 0.818 | 0.333 | 0.912 | 0.735 | 0.581 | 0.824 | 0.576 | 0.879 | 0.576 | 0.879 | 0.594 | 0.658 |
|  | *H_E_* | 0.733 | 0.750 | 0.867 | 0.806 | 0.813 | 0.741 | 0.692 | 0.773 | 0.636 | 0.858 | 0.641 | 0.851 | 0.672 | 0.756 |
|  | *F_IS_* | 0.808 | 0.059 | 0.057 | 0.59 | -0.123 | 0.008 | 0.163 | -0.067 | 0.096 | -0.025 | 0.103 | -0.033 | 0.118 | 0.135 |
|  | *P_HW_* | 0.000 | 0.177 | 0.018 | 0.000 | 0.132 | 0.427 | 0.023 | 0.309 | 0.306 | 0.419 | 0.374 | 0.904 | 0.075 | — |
| 6 | *A/A_r_* | 11/9.0 | 10/9.1 | 9/8.7 | 9/8.1 | 8/7.1 | 15/12.0 | 9/8.2 | 9/8.7 | 13/11.5 | 13/12.7 | 12/9.6 | 9/8.0 | 12/10.3 | 10.7/9.5 |
| *N* = 48 | *H_O_* | 0.478 | 0.739 | 0.600 | 0.546 | 0.522 | 0.717 | 0.217 | 0.761 | 0.804 | 0.841 | 0.783 | 0.750 | 0.778 | 0.657 |
|  | *H_E_* | 0.523 | 0.728 | 0.806 | 0.828 | 0.813 | 0.726 | 0.720 | 0.835 | 0.802 | 0.914 | 0.851 | 0.699 | 0.856 | 0.777 |
|  | *F_IS_* | 0.087 | -0.016 | 0.258 | 0.344 | 0.361 | 0.012 | 0.7 | 0.089 | -0.003 | 0.081 | 0.081 | -0.074 | 0.093 | 0.155 |
|  | *P_HW_* | 0.220 | 0.111 | 0.193 | 0.000 | 0.000 | 0.829 | 0.000 | 0.220 | 0.045 | 0.100 | 0.542 | 0.371 | 0.367 | — |
| 7 | *A/A_r_* | 4/3.8 | 14/12.9 | 8/7.9 | 7/6.9 | 7/6.5 | 6/5.3 | 8/7.7 | 6/5.5 | 8/6.6 | 14/12.1 | 9/8.2 | 11/9.3 | 9/6.4 | 8.5/7.6 |
| *N* = 45 | *H_O_* | 0.311 | 0.778 | 0.535 | 0.707 | 0.727 | 0.422 | 0.257 | 0.533 | 0.533 | 0.857 | 0.844 | 0.800 | 0.578 | 0.606 |
|  | *H_E_* | 0.408 | 0.908 | 0.858 | 0.825 | 0.783 | 0.501 | 0.821 | 0.491 | 0.617 | 0.840 | 0.790 | 0.785 | 0.631 | 0.712 |
|  | *F_IS_* | 0.239 | 0.145 | 0.38 | 0.144 | 0.072 | 0.159 | 0.69 | -0.088 | 0.137 | -0.02 | -0.07 | -0.019 | 0.086 | 0.143 |
|  | *P_HW_* | 0.030 | 0.001 | 0.000 | 0.118 | 0.560 | 0.186 | 0.000 | 0.832 | 0.174 | 0.088 | 0.101 | 0.241 | 0.506 | — |
| 8 | *A/A_r_* | 10/7.6 | 17/14.2 | 12/9.6 | 13/12.1 | 8/7.4 | 19/12.6 | 19/15.6 | 11/10.6 | 19/15.6 | 22/18.2 | 11/10.2 | 13/10.7 | 13/11.2 | 14.4/12.0 |
| *N* = 50 | *H_O_* | 0.200 | 0.780 | 0.500 | 0.680 | 0.800 | 0.700 | 0.400 | 0.900 | 0.800 | 0.920 | 0.940 | 0.720 | 0.780 | 0.702 |
|  | *H_E_* | 0.693 | 0.915 | 0.864 | 0.841 | 0.806 | 0.716 | 0.913 | 0.862 | 0.918 | 0.939 | 0.835 | 0.707 | 0.855 | 0.836 |
|  | *F_IS_* | 0.690 | 0.149 | 0.408 | 0.193 | 0.007 | 0.022 | 0.522 | -0.045 | 0.130 | 0.020 | -0.127 | -0.019 | 0.088 | 0.157 |
|  | *P_HW_* | 0.000 | 0.002 | 0.000 | 0.070 | 0.107 | 0.275 | 0.000 | 0.307 | 0.064 | 0.133 | 0.312 | 0.830 | 0.000 | — |

| Sites |  | | RCM  S007 | | RCM  S009 | | RCM  S010 | | RCM  S011 | | RCM  S026 | | RCM  S028 | | RCM  S029 | | RCM  S030 | | | RCM  S035 | | RCM  S042 | | RCM  S092 | | RCM  S098 | | RCM  S107 | Average |
| --- | --- | --- | --- | --- | --- | --- | --- | --- | --- | --- | --- | --- | --- | --- | --- | --- | --- | --- | --- | --- | --- | --- | --- | --- | --- | --- | --- | --- | --- |
| 9 | | *A/A_r_* | | 8/5.9 | | 19/16.1 | | 12/9.7 | | 10/9.2 | | 9/7.0 | | 16/10.4 | | 13/10.6 | | 11/9.7 | 18/14.2 | | 18/15.3 | | 12/11.1 | | 16/11.1 | | 11/9.7 | | 13.3/10.8 |
| *N* = 50 | | *H_O_* | | 0.220 | | 0.860 | | 0.700 | | 0.860 | | 0.780 | | 0.520 | | 0.280 | | 0.800 | 0.720 | | 0.860 | | 0.900 | | 0.640 | | 0.660 | | 0.677 |
|  | | *H_E_* | | 0.644 | | 0.919 | | 0.847 | | 0.845 | | 0.794 | | 0.577 | | 0.824 | | 0.826 | 0.868 | | 0.924 | | 0.887 | | 0.639 | | 0.815 | | 0.801 |
|  | | *F_IS_* | | 0.575 | | 0.065 | | 0.152 | | -0.018 | | -0.014 | | 0.099 | | 0.578 | | 0.032 | 0.172 | | 0.026 | | -0.014 | | -0.047 | | 0.192 | | 0.138 |
|  | | *P_HW_* | | 0.000 | | 0.232 | | 0.099 | | 0.701 | | 0.871 | | 0.169 | | 0.000 | | 0.169 | 0.000 | | 0.260 | | 0.523 | | 0.890 | | 0.028 | | — |
| 10 | | *A/A_r_* | | 5/4.8 | | 11/10.9 | | 5/4.7 | | 8/8.0 | | 8/7.6 | | 7/6.9 | | 9/8.5 | | 11/9.9 | 9/8.3 | | 9/8.6 | | 10/8.9 | | 11/10.4 | | 9/8.7 | | 8.6/8.2 |
| *N* = 32 | | *H_O_* | | 0.500 | | 0.875 | | 0.531 | | 0.321 | | 0.813 | | 0.742 | | 0.129 | | 0.719 | 0.563 | | 0.750 | | 0.813 | | 0.774 | | 0.400 | | 0.610 |
|  | | *H_E_* | | 0.560 | | 0.901 | | 0.572 | | 0.844 | | 0.801 | | 0.677 | | 0.766 | | 0.803 | 0.771 | | 0.838 | | 0.783 | | 0.847 | | 0.819 | | 0.768 |
|  | | *F_IS_* | | 0.109 | | 0.03 | | 0.072 | | 0.623 | | -0.015 | | -0.098 | | 0.834 | | 0.106 | 0.274 | | 0.107 | | -0.038 | | 0.087 | | 0.516 | | 0.201 |
|  | | *P_HW_* | | 0.257 | | 0.039 | | 0.699 | | 0.000 | | 0.5215 | | 0.404 | | 0.000 | | 0.705 | 0.407 | | 0.034 | | 0.396 | | 0.045 | | 0.000 | | — |
| 11 | | *A/A_r_* | | 3/2.8 | | 9/8.8 | | 8/7.3 | | 3/3.0 | | 3/2.9 | | 2/2.0 | | 2/1.8 | | 4/3.4 | 5/4.3 | | 4/4.0 | | 4/3.9 | | 2/2.0 | | 8/7.2 | | 4.4/4.1 |
| *N* = 50 | | *H_O_* | | 0.200 | | 0.833 | | 0.489 | | 0.082 | | 0.939 | | 0.449 | | 0.000 | | 0.306 | 0.580 | | 0.760 | | 0.306 | | 0.320 | | 0.457 | | 0.200 |
|  | | *H_E_* | | 0.185 | | 0.858 | | 0.826 | | 0.511 | | 0.542 | | 0.488 | | 0.040 | | 0.391 | 0.584 | | 0.707 | | 0.339 | | 0.298 | | 0.779 | | 0.504 |
|  | | *F_IS_* | | -0.082 | | 0.029 | | 0.410 | | 0.842 | | -0.745 | | 0.081 | | 1.000 | | 0.219 | 0.007 | | -0.076 | | 0.098 | | -0.074 | | 0.417 | | 0.164 |
|  | | *P_HW_* | | 1.000 | | 0.883 | | 0.000 | | 0.000 | | 0.000 | | 0.767 | | 0.010 | | 0.190 | 0.473 | | 0.305 | | 0.056 | | 1.000 | | 0.000 | | — |
| 12 | | *A/A_r_* | | 2/2.0 | | 11/10.2 | | 9/7.8 | | 4/3.5 | | 3/3.0 | | 12/10.8 | | 10/8.8 | | 4/3.5 | 11/10.7 | | 10/8.1 | | 6/5.9 | | 10/9.0 | | 7/5.3 | | 7.6/6.8 |
| *N* = 50 | | *H_O_* | | 0.333 | | 0.760 | | 0.447 | | 0.213 | | 0.872 | | 0.792 | | 0.08 | | 0.265 | 0.222 | | 0.523 | | 0.857 | | 0.936 | | 0.604 | | 0.531 |
|  | | *H_E_* | | 0.281 | | 0.832 | | 0.693 | | 0.252 | | 0.616 | | 0.831 | | 0.714 | | 0.306 | 0.855 | | 0.665 | | 0.796 | | 0.739 | | 0.618 | | 0.631 |
|  | | *F_IS_* | | -0.19 | | 0.087 | | 0.358 | | 0.157 | | -0.423 | | 0.047 | | 0.889 | | 0.133 | 0.743 | | 0.216 | | -0.077 | | -0.273 | | 0.022 | | 0.130 |
|  | | *P_HW_* | | 0.323 | | 0.076 | | 0.000 | | 0.156 | | 0.000 | | 0.375 | | 0.792 | | 0.095 | 0.000 | | 0.122 | | 0.183 | | 0.048 | | 0.828 | | — |
