# Supplementary material for: Big mountains but small barriers: Population genetic structure of the Chinese wood frog (Rana chensinensis) in the Tsinling and Daba Mountain region of northern China
Source: BMC Genet. 2009 Apr 9;10:17. doi: 10.1186/1471-2156-10-17 (PMC2679764; doi:10.1186/1471-2156-10-17)
Supplement: Additional File 2 — Estimates of population genetic differentiation (pairwise FST) and migration rate between sites. The file provided the original data of the pairwise FST and estimates of numbers of immigrants and numbers of emigrants between sites. [file 1471-2156-10-17-S2.doc]

**Additional file 2**

Estimates of population genetic differentiation (pairwise *F_ST_*; below diagonal) and migration rate (*N_m_*; above diagonal) between sites. Pairwise *F_ST_* was estimated using ARLEQUIN, and all are statistically significant. *N_m_* was estimated using MIGRATE, and the numbers out of the brackets are numbers of immigrants received by populations in the left-most column and the numbers in the brackets are numbers of emigrants moving out of populations in the left-most column.

|  | North of Tsinling | | | | | South of Tsinling/North of Daba | | | | South of Daba | | |
| --- | --- | --- | --- | --- | --- | --- | --- | --- | --- | --- | --- | --- |
| Sites | 1 | 2 | 3 | 4 | 5 | 6 | 7 | 8 | 9 | 10 | 11 | 12 |
| 1 | ***** | 3.24(2.30) | 0.96(0.61) | 0.88(0.69) | 1.33(0.97) | 0.48(0.53) | 0.17(0.32) | 0.38(0.46) | 0.79(0.58) | 0.73(0.41) | 0.11(0.41) | 0.22(0.74) |
| 2 | 0.0175 | ***** | 0.58(0.67) | 0.96(0.73) | 0.57(1.57) | 0.42(0.46) | 0.95(0.23) | 0.67(0.53) | 0.82(0.62) | 0.13(1.46) | 0.19(0.64) | 0.11(0.24) |
| 3 | 0.0740 | 0.0770 | ***** | 1.09(1.17) | 0.94(0.69) | 0.66(0.96) | 0.77(0.93) | 1.26(0.51) | 1.05(0.53) | 1.00(1.17) | 0.64(0.88) | 0.87(0.91) |
| 4 | 0.0777 | 0.0841 | 0.0510 | ***** | 0.47(0.62) | 1.17(0.54) | 1.11(0.39) | 0.64(0.79) | 1.00(0.92) | 1.08(0.89) | 0.77(0.42) | 1.20(0.77) |
| 5 | 0.0772 | 0.0803 | 0.0826 | 0.0858 | ***** | 0.88(0.47) | 1.13(0.51) | 0.66(0.90) | 0.76(0.77) | 1.04(0.48) | 0.11(0.47) | 0.35(0.68) |
| 6 | 0.1050 | 0.0991 | 0.0964 | 0.0667 | 0.0845 | ***** | 0.98(0.86) | 1.53(1.00) | 0.79(1.15) | 0.81(0.58) | 0.97(0.76) | 0.91(1.06) |
| 7 | 0.1606 | 0.1625 | 0.1261 | 0.1171 | 0.1497 | 0.0906 | ***** | 0.79(0.91) | 0.73(0.86) | 0.71(0.70) | 0.21(0.52) | 0.19(0.91) |
| 8 | 0.0838 | 0.0924 | 0.0670 | 0.0473 | 0.0895 | 0.0658 | 0.0837 | ***** | 1.21(0.99) | 1.03(1.02) | 0.75(0.38) | 0.89(0.35) |
| 9 | 0.1047 | 0.1138 | 0.0879 | 0.0585 | 0.1089 | 0.0580 | 0.0773 | 0.0217 | ***** | 0.44(0.57) | 1.03(0.39) | 0.93(0.85) |
| 10 | 0.1056 | 0.1128 | 0.0909 | 0.0807 | 0.1138 | 0.0866 | 0.1257 | 0.0517 | 0.0588 | ***** | 0.12(1.05) | 0.71(1.07) |
| 11 | 0.2536 | 0.2700 | 0.2352 | 0.2291 | 0.2801 | 0.2607 | 0.3130 | 0.2424 | 0.2454 | 0.2490 | ***** | 0.40(0.20) |
| 12 | 0.1606 | 0.1961 | 0.1528 | 0.1327 | 0.1922 | 0.1837 | 0.2356 | 0.1272 | 0.1533 | 0.1736 | 0.2663 | ***** |
